# *In vitro* and *in silico* characterization of alkaline serine protease from *Bacillus subtilis* D9 recovered from Saudi Arabia

**DOI:** 10.1016/j.heliyon.2021.e08148

**Published:** 2021-10-08

**Authors:** Amal Mahmoud, Essam Kotb, Amany I. Alqosaibi, Ahmed A. Al-Karmalawy, Ibtesam S. Al-Dhuayan, Hameedah Alabkari

**Affiliations:** a Department of Biology, College of Science, Imam Abdulrahman Bin Faisal University, P.O. Box 1982, 31441, Dammam, Saudi Arabia; b Basic & Applied Scientific Research Center, Imam Abdulrahman Bin Faisal University, P.O. Box 1982, 31441, Dammam, Saudi Arabia; c Department of Pharmaceutical Medicinal Chemistry, Faculty of Pharmacy, Horus University-Egypt, New Damietta 34518, Egypt

**Keywords:** Alkaline serine protease gene, Phylogenetic analysis, *B. subtilis*, *B. cereus*, Subtilase domain, Catalytic triad

## Abstract

In this study, we have isolated and characterized proteolytic soil bacteria and their alkaline protease. Based on 16S rRNA sequence analysis, 12 isolates with the highest protease activity were classified as *B. subtilis* and *B. cereus* groups. *B. subtilis* D9 isolate showing the highest protease activity was selected for *in vitro* and *in silico* analysis for its ِِAKD9 protease. The enzyme has a molecular mass of 48 kDa, exhibiting optimal activity at 50 °C pH 9.5, and showed high stability till 65 °C and pH 8–11 for 1 h. Fe^3+^‏ stimulated, but Zn^2+^ and Hg^2+^ strongly inhibited the protease activity. Also, the maximum inhibition with PMSF indicated serine protease-type of AKD9 protease. AkD9 alkaline serine protease gene showed high sequence similarity and close phylogenetic relationship with AprX serine protease of *B. subtilis* isolates. Functional prediction of AKD9 resulted in the detection of subtilase domain, peptidase_S8 family, and subtilase active sites. Moreover, prediction of physicochemical properties indicated that AKD9 serine protease is hydrophilic, thermostable, and alkali-halo stable. Secondary structure prediction revealed the dominance of the coils enhances AKD9 activity and stability under saline and alkaline conditions. Based on molecular docking, AKD9 showed very promising binding affinities towards casein substrate with expected intrinsic proteolytic activities matching our obtained *in vitro* results. In conclusion, AKD9 alkaline serine protease seems to be a significant candidate for industrial applications because of its stability, hydrophilicity, enhanced thermostability, and alkali-halo stability.

## 1 Introduction

As one of the main industrial enzymes, proteases are responsible for around 60% of the world enzyme market (Clarridge, 2004; Raveendran et al., 2018; Razzaq et al., 2019). However, serine proteases represent one-third of the share in the enzyme market (Page and Di Cera, 2008). Microbial proteases are preferred over animal and plant proteases due to their easy genetic manipulation and wide biochemical diversity (Chanalia et al., 2011). A wide variety of organisms produce alkaline proteases. *Bacillus sp.* are mostly the microbial source to generate the proteases. *Bacillus*-derived alkaline proteases have numerous applications in industry, including organic synthesis, food, pharmacology, leather, and bioremediation (Tarhriz et al., 2014; Al-Dhuayan et al., 2021).

The functional groups in the proteases’ active sites are classified as aspartic, cysteine, metallo, glutamic, serine, and threonine proteases. In addition, they are classified into exo and endo-peptidases based on the position of the peptide bond cleavage. Also, proteases are classified into three types based on their maximum activity in a wide range of pH, including alkaliphile, neutral, and acidophile proteases (Pushpam et al., 2011; Fath and Fazaelipoor, 2015). Furthermore, factors such as substrate specificity, enzyme action, amino acid residue similarity in the active site, and catalytic activity mechanism play a role in protease activity (Ellaiah et al., 2002). Based on their structure, serine proteases are classified into chymotrypsin-like (trypsin-like) or subtilisin-like (Madala et al., 2010). Subtilisin or subtilase family (Peptidase family S8) is the second-largest family of serine proteases. Proteases in this family are characterized by an Asp/His/Ser catalytic triad (Rawlings et al., 2006, 2010).

Proteases of *Bacillus* species are *in vitro* characterized by molecular weight range 27–71 kDa, optimal pH range 6–10, temperature 37 ºC–60 °C, and stability values over a wide range of pH values and temperatures (Karray et al., 2021). Moreover, *in silico* characterization of alkaline proteases would help understand their industrial application and large-scale production (Zhang et al., 2015; Dash et al., 2017; Saggu and Mishra, 2017; Zhou et al., 2018).

The structure of proteases, catalytic mechanism, and specificity are essential factors in enzyme application that should be considered (Morya et al., 2012). The protein structure prediction tools are appreciated in studying protein structures and function (Marks et al., 2012; Elhefnawi et al., 2019). Different attempts have been made to understand the binding efficiency of the modeled protease from *Bacillus* sp. and different substrates by molecular docking studies (Baweja et al., 2016; Kandasamy et al., 2017).

This study reveals a new alkaline serine protease's biochemical properties, catalytic potentials, and binding patterns using *in vitro* and *in silico* techniques.

## 2 Materials and methods

Ninety-three soil samples were collected from the Eastern Province (Dhahran) and Riyadh (Shaqra) of Saudi Arabia and were used for bacterial screening and production of their alkaline proteases.

### 2.1 Isolation and screening for alkaline protease producing bacteria

All bacterial isolates were initially screened for their proteolytic activity onto alkaline casein agar (pH 9.4) involving (g/L): Agar (15), peptone (5), casein (5), and yeast extract (1). Incubation was done for 24 h at 40 °C. Next, proteolytic bacterial isolates were purified through quadrate streaking onto nutrient agar Petri dishes (pH 9.0) composed of (g/L): Agar (15), peptone (5), sodium chloride (5), meat extract (1), and yeast extract (2). Recovered bacterial isolates were then preserved into 20% (v/v) glycerol at -80 ^○^C.

### 2.2 Enzyme production

#### 2.2.1 Qualitative screening of the enzyme

Bacterial isolate D9 was selected for further microbiological and biochemical investigation, as it produced the highest clearance zone around its growing colonies on casein agar. The qualitative screening of the alkaline protease (AKD9) of isolate D9 was carried out on the basal medium described by Kotb (2015) containing (g/L): Soybean powder (5), lactose sugar (10), K_2_HPO_4_ (0.5), KH_2_PO_4_ (1.5), CaCl_2_ (2), and MgSO_4_.7H_2_O (0.5). The growth was allowed for 48 h at 40 °C in a 50 mL medium (pH 9.5). Inoculation was done using 1% (v/v) of an overnight bacterial broth having A_600_ of about 0.2 and incubated in an orbital shaker at the rate of 120 rpm.

#### 2.2.2 Protein quantification and protease activity assay

Protein quantification was measured directly at A_280_. Protease activity was evaluated by mixing 1 ml of the enzyme with 1 ml of 1.0% (w/v) casein dissolved in 0.1 M glycine–NaOH buffer, pH 10.0. The enzyme-substrate mixtures were incubated at 45 °C for 30 min. The reaction was terminated by adding 2 ml of 10% (w/v) trichloroacetic acid stop solution. The A_280_ of the supernatant was measured and converted to tyrosine equivalents. One unit of protease activity (U) is defined as the quantity of protease releasing one micromole of equivalent L-tyrosine/min under the standard conditions of the assay.

#### 2.2.3 Enzyme purification

After producing AKD9 alkaline protease under the aforementioned conditions, the bacterial culture supernatant was fractionated by applying 30–60% (NH_4_)_2_SO_4_ precipitation. The resulting proteins were collected by centrifugation for 20 min at a speed of 10,000*g* and resuspended in 6 ml of 20 mM borate buffer (pH-8.5). The first elution of the crude enzyme was done through the DEAE-Sepharose CL-6B column (2.5 × 30 cm^2^) equilibrated with the typical buffer. Next, the unbounded proteins were eluted at a 1 ml/min rate with the same buffer with a linear rise in the ionic strength from 20 mM to 800 mM. Protease active fractions were then concentrated and subjected to the second elution through Sephadex G-100 FF column (2 × 70 cm^2^) at a 0.5 ml/min rate using 20 mM borate buffer (pH-7.4). The final potent protease fractions were lyophilized and analyzed for enzymatic purity by SDS-PAGE analysis, according to Andrews (1986), using 10% resolving gel and 4% stacking gel with a constant volt of 60V.

### 2.3 Enzyme characterization

#### 2.3.1 Effect of temperature on enzyme activity and stability

The effect of temperature on the AKD9 alkaline protease activity was analyzed at a varied temperature range (30–80 °C). The thermal stability was checked by pre-incubating the enzyme for 60 min at the same range of temperatures, followed by measuring their percent of residual activities against the substrate.

#### 2.3.2 Effect of pH on enzyme activity and stability

The effect of pH on the enzymatic activity was assessed using 0.1 M buffer systems comprising phosphate buffer (pH 6–7), Tris-HCl buffer (pH 8–9), and glycine–NaOH buffer (pH 10–12). The pH stability analysis was conducted prior to the incubation of the protease enzyme for 1 h at 35 °C in the same buffers (pH 6–12), and residual activities were analyzed.

Isoelectric point (p*I*) was defined as the pH value at which maximum protein was measured. Concentrated fractions of AKD9 protease were incubated for 12 h at a pH range of 3.0–10.8 at 4 °C using the buffer systems mentioned above to determine the p*I* of the protease. In each separate test, precipitated proteins after centrifugation at 10,000 *g* for 15 min were quantified by resuspending in 1ml Tris-HCl buffer (pH 7.5). Then, the absorbances A_280_ and A_260_ were measured applying the following equation: Soluble protein (mg ml^−1^) = 1.55 OD_280_ - 0.76 OD_260_.

#### 2.3.3 Effects of metal ions and protease inhibitors on the purified protease

The effect of various metal ions on the activity of the purified AkD9 protease was investigated by adding metal ions (Fe ^2+^, Ca^2+^, Mn^2+^, Zn^2+^, Cu^2+^, Ba^2+^, Mg^2+^, and Hg^2+^) to the enzyme and incubated for 1 h. The effect of protease inhibitors was also assessed using EDTA, PMSF, SBTI, TLCK, aprotinin, 2,2′-bipyridine, and *o*-phenanthroline. The activity of the enzyme in the absence of metal ions and inhibitors was taken as 100% activity.

### 2.4 *In silico* analysis of the bacterial isolates and its alkaline protease gene

Bacterial isolates showing the maximum alkaline protease productivity were selected for *in silico* identification and characterization using 16S rRNA and alkaline protease gene.

#### 2.4.1 PCR amplification of 16S rRNA and protease genes

Twelve soil bacterial isolates (D2, D9, D10, D14, D26, D30, D35, D40, D42, D44, D46, and D48) showing the maximum alkaline protease productivity were used for amplification of the 16S rRNA gene. PCR was done directly from the bacterial glycerol stock according to Macrogen. Primers F 5′AGA-GTTTGATCCTGGCTCAG-3′ and R 5′-GGTTACCTTGTTACGACTT-3′ were used for PCR amplification of the 16S rRNA gene (Srinivasan et al., 2015). Isolate D9 with high protease activity was used for amplification of alkaline protease gene AKD9. Alkaline protease primers F 5′ CATATGTTTGGGTACTCTATGG-3′ and R 5′ GGATCCTTATTGGCCGGGAACGGAA-3′ were used (Sadeghi et al., 2009). PCR was performed according to the manufacture of PCR SuperMix Invitrogen™. The PCR product containing alkaline protease gene was extracted from 0.8% agarose gel using QIAquick Gel Extraction Kit (Qiagen), and inserted into TA Cloning® Kit, with pCR™2.1 Vector, Invitrogen. The recombinant plasmid was transformed into DH5α *E. coli* cells.

#### 2.4.2 Sequence analysis

The 16S rRNA and alkaline protease products were sent to Macrogen for sequencing, sequences were edited using Chromas version 2.6.6 (https://technelysium.com.au/wp/chromas/); sequence identity and similarity were performed through BLAST programs from National Center for Biotechnology Information (NCBI), USA (http://www.ncbi.nlm.nih.gov/Blast). In addition, 16S rRNA and alkaline protease sequences of different bacterial isolates were retrieved from the BLAST-NCBI database for multiple sequence alignment and phylogenetic tree construction.

#### 2.4.3 GenBank submission

The 16S rRNA and alkaline protease sequences were submitted to GenBank under the accession numbers MK819971–MK819982 for the 16S rRNA gene and MK814958 (protein_id QGA88715.1) for the AKD9 alkaline protease gene.

#### 2.4.4 Multiple sequence alignment and phylogenetic analysis

Molecular evolutionary genetics analysis (MEGA) software version MEGA 6.0 was used for multiple sequence alignment analysis, neighbor-joining phylogenetic trees construction, and bootstrap analysis (Tamura et al., 2013).

#### 2.4.5 Prediction of functional sites and protein family

Expasy-PROSITE tools (https://prosite.expasy.org/) are protein databases for identifying protein domains, families, and functional sites as well as associated patterns and profiles (Sigrist et al., 2013). ScanProsite (https://prosite.expasy.org/scanprosite/) is one of the Expasy-PROSITE tools that was used to predict the catalytic domain and the active sites of AKD9 protease.

Also, Pfam (http://pfam.sanger.ac.uk/) is a database of conserved protein families and domains, Pfam is a member database of InterPro (http://www.ebi.ac.uk/interpro/about/interpro) as part of EMBL-EBI (https://www.ebi.ac.uk). Pfam database was used to identify the AkD9 protein family.

#### 2.4.6 Physicochemical properties

The physical and chemical attributes, such as molecular weight, theoretical p*I*, amino acid composition, atomic composition, instability index, aliphatic index, and grand average of hydropathy (GRAVY), of the AKD9 protease were computed using the ProtParam assessment tool of the ExPASy server (http://web.expasy.org/protparam/).

#### 2.4.7 Subcellular localization

The subcellular location of the alkaline protease AKD9 was documented by utilizing the PSORTb v.3.0.2 (https://www.psort.org/psortb/) (Yu et al., 2010).

#### 2.4.8 Secondary structure prediction

The self-optimized prediction method with alignment (SOPMA) tool (Geourjon and Deleage, 1995) was used for predicting the secondary structure of AKD9 protease (https://npsa-prabi.ibcp.fr/cgi-bin/npsa_automat.pl?page=/NPSA/npsa_sopma.html). The method works by making consensus predictions from multiple alignments. The positional possibility of alpha helix, beta sheets, beta turns, and random coils was assessed using the default parameters of this tool.

#### 2.4.9 Protein-protein docking studies

The protein-protein docking process for AKD9 alkaline serine protease isolated from *B. subtilis* and casein substrate was performed using MOE 2019 drug design suite (Inc, 2016). It was carried out to describe and evaluate the binding affinity of casein towards alkaline serine protease and confirm its proteolytic potential in a 3D manner.

#### 2.4.10 Preparation of target proteins

The Protein Data Bank (https://www.rcsb.org/) was used to extract the X-ray structures of both the alkaline serine protease and casein (PDB codes: 1WMD (Nonaka et al., 2004) and 1QF8 (Chantalat et al., 1999), respectively). Protein preparation protocol was applied for the preparation of the proteins where automatic correction for any errors in the connections and types of atoms, the addition of hydrogen atoms in 3D geometry, and energy minimization in keeping all atoms in the free movement were applied as previously described (Abo Elmaaty et al., 2021; Samra et al., 2021).

#### 2.4.11 Protein-protein docking process

The protein-protein docking protocol was selected for describing the interactions between alkaline serine protease and casein. The applied methodology was as follows: The previously prepared alkaline serine protease protein (1WMD) was inserted in the place of the receptor; the prepared casein protein was selected to be the ligand; the hydrophobic patch potential for the ligand site was selected before initiating the docking process. The scoring tools were adjusted to default values. The number of pre-placement poses, placement poses, and refinement poses were 10000, 1000, and 100, respectively (El-Shal et al., 2021). The obtained 100 poses by the end of the docking process were screened to select one showing the best protein-protein interactions and score value.

## 3 Results and discussion

### 3.1 Isolation and preliminary screening of bacterial isolates for the protease activity

Of 93 soil bacterial isolates collected from the Eastern Province (Dhahran) and Riyadh (Shaqra) of Saudi Arabia, 12 bacterial isolates have showed the maximum alkaline protease activity upon preliminary screening. Among these, isolate D9 formed a remarkable hydrolytic zone of clearance around its colonies and therefore, was chosen for further study.

### 3.2 Molecular identification of the alkaline protease-producing bacteria

**Figure 1:**
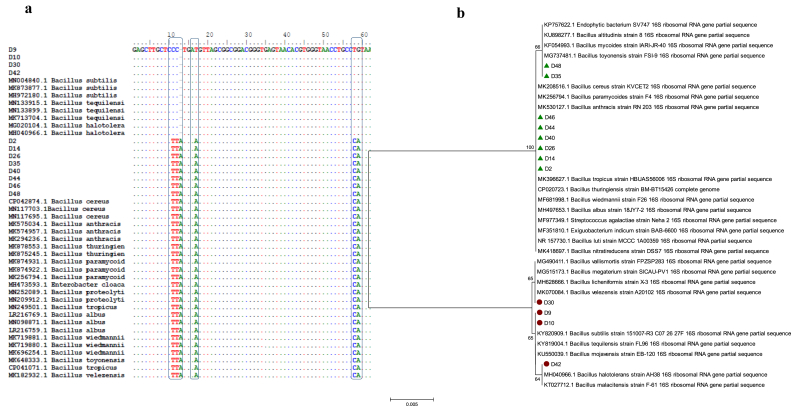
Molecular identification of the alkaline protease-producing bacteria based on its 16S rRNA gene sequence showing multiple sequence alignment of 16S rRNA gene, the 16S rRNA alterations are boxed (a) and Neighbor-joining phylogenetic analysis of 16S rRNA gene (b).

The 12 high alkaline protease-producing bacterial isolates were identified through amplification and sequence analysis of the 16S rRNA gene. BLASTn, multiple sequence alignment, and phylogenetic analysis of 16S rRNA sequence (Figure 1a and b) showed that isolates D9, D10, D30, and D42 belong to the *B. subtilis* group (99.89%–100%). However, isolates D2, D14, D26, D35, D40, D44, D46, and D48 belong to the *B. cereus* group (99.79%–100%). It was noticed that five nucleotide substitutions and one indel (C11→T, C12→T, '-'13→A, T17→A, T58→C and G59→A) differentiated between *B. subtilis* and *B. cereus* groups (Figure 1a). Although our isolates were classified, at the group level, as members of *B. subtilis* and *B. cereus* groups, the high identity (99.8%–100%) between our isolates and those of *B. subtilis* and *B. cereus* groups prevented the classification at the species level. Some researchers indicated that the highly similar sequences of the 16S rRNA gene make the identification of some *Bacillus* species difficult (Maughan and Van der Auwera, 2011; Mohkam et al., 2016).

### 3.3 Purification and molecular weight determination of protease from D9 isolate

**Figure 2:**
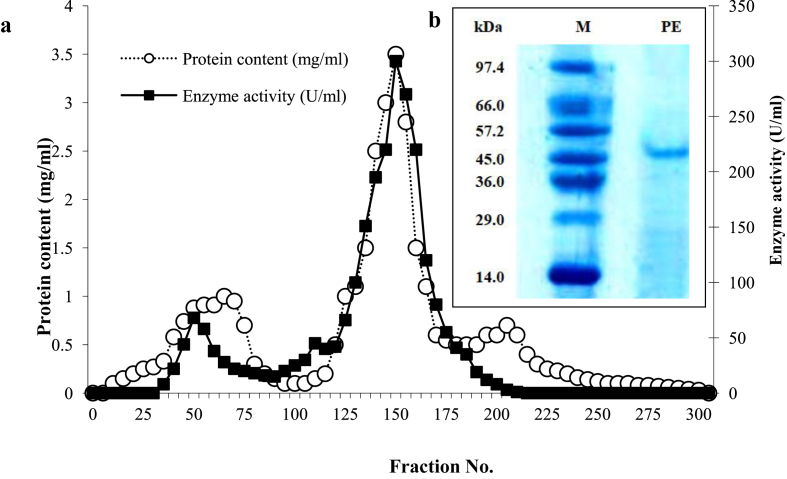
Elution profile of the purified protease from isolate D9 through Sephadex G-100 (a) and SDS-PAGE (b). Lane M was loaded with the marker proteins, while lane PE was loaded with the purified AKD9 protease.

Based on the potency in productivity, the alkaline protease AKD9 of *B. subtilis* isolate D9 was selected for enzyme purification and biochemical characterization. The protein in the final active fractions obtained from Sephadex G-100 elution (Figure 2a) was analyzed by SDS-PAGE, which declared the presence of a single band just below 48 kDa (Figure 2b), lying within the molecular weight range (27–71 kDa). This was reported for most of the alkaline proteases isolated from *Bacillus* species (Karray et al., 2021).

### 3.4 Enzyme characterization

#### 3.4.1 Effect of temperature on enzyme activity and stability

**Figure 3:**
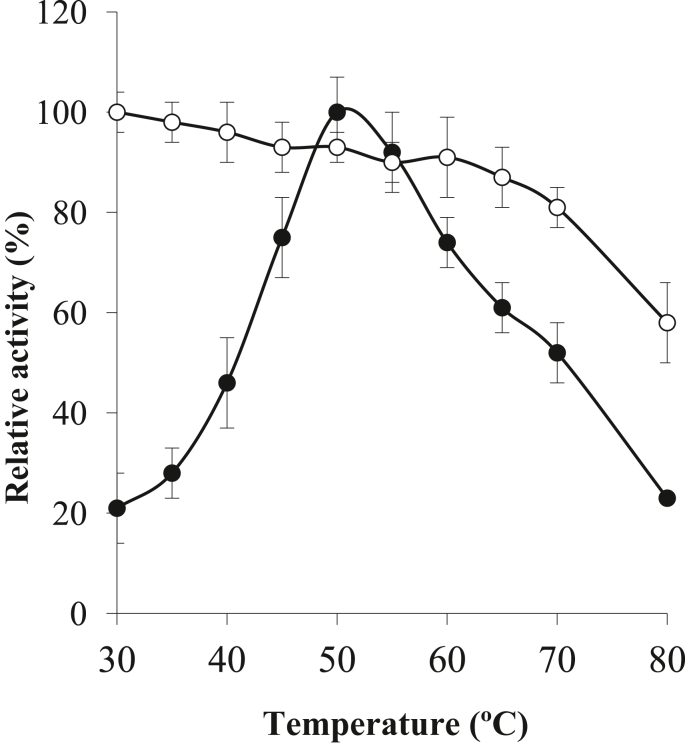
Effect of temperature upon the AkD9 protease activity (-●-) and stability (-○-).

Temperature is considered as one of the essential factors influencing both protease activity and stability. The temperature optima and thermostability vary based on the bacterial species. AKD9 protease exhibited optimum activity at temperature 50 °C and stability till 65 °C (Figure 3), which coincides with the proteases involved in the commercial detergents like Surf, Henko, Ariel, Mr. White, and Rin. It also had the maximum protease activity at 50 °C and showed poor protease activity at 40 °C and very low activity at 60 °C (Sharma et al., 2019).

#### 3.4.2 Effect of pH on enzyme activity and stability

**Figure 4:**
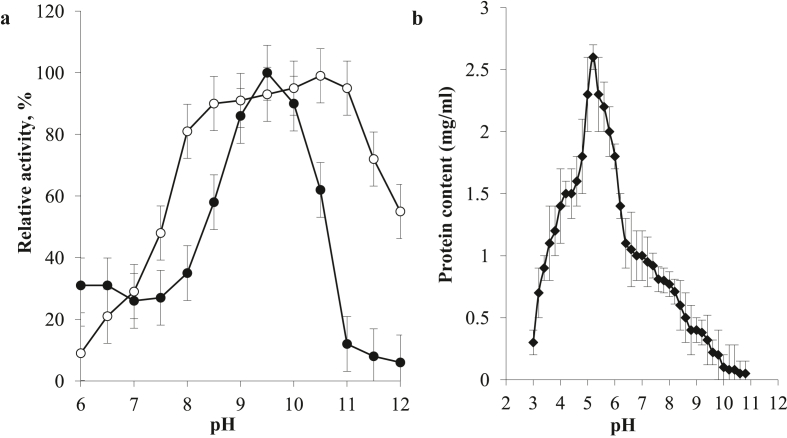
Effect of pH upon the AkD9 protease activity (-●-) and stability (-○-) (a) and isoelectric point based on pH precipitation profile (b).

The changes in the pH values affecting both protease activity and stability were also tested. The highest protease activity (100%) was observed at pH 9.5. Interestingly, we noticed that the purified enzyme was highly stable in the pH range of 8–11 after 1 h of incubation without the reacting substrate (Figure 4a). Moreover, the isoelectric point (p*I*) was found at pH 5.2 (Figure 4b). In other investigations, alkaline proteases from *Aureobasidium pullulans*, *Yarrowia lipolytica*, *Issatchenkia orientalis,* and *Cryptococcus aureus* had an optimum pH of 9–10 and optimum temperature of 45–50 °C (Li et al., 2009).

#### 3.4.3 Effect of metal ions on the enzyme activity

Metal ions play an important role in maintaining the active conformation of proteases (Sharma et al., 2019). The influence of metals and protease inhibitors on the activity of AKD9 was performed (Table 1). The pure form of the AKD9 enzyme was inhibited in the presence of Hg^2+^ (residual activity = 17.2 U/ml) and Zn^2+^(residual activity = 21.3 U/ml). However, Fe^3+^ ions exerted the highest stimulatory effect (residual activity = 129.0 U/ml). Mn^2+^ showed a very low stimulatory effect (102.5 U/ml). Referring to literature, Paul et al. (2011) reported that alkaline serine protease requires Co^2+^ or Mn^2+^ metal ions to improve activity.

**Table 1:** Effect of metal ions and protease inhibitors on AKD9 enzyme activity.

Inhibitors or metal ions	Concentration, mM	Residual activity, U/ml
None	-	100.5 ± 1.5
ZnCl_2_	5	21.3 ± 1.7
CaCl_2_	5	101.5 ± 2.0
BaCl_2_	5	95.5 ± 0.6
FeCl_3_	5	129.0 ± 2.1
CuCl_2_	5	88.9 ± 1.5
MgCl_2_	5	98.6 ± 1.4
HgCl_2_	5	17.2 ± 0.5
MnCl_2_	5	102.5 ± 2.1
EDTA	5	101.5 ± 1.1
2,2′-Bipyridine	0.1	98.8 ± 1.3
TLCK	0.1	31.5 ± 0.4
Aprotinin	0.1	25.7 ± 0.3
*o*-Phenanthroline	0.1	97.7 ± 1.0
PMSF	10	1.5 ± 0.1
SBTI	0.1	7.2 ± 0.2

According to Table 1, the metalloprotease inhibitors had little effect on the enzymatic activity. However, the serine protease inhibitors, especially PMSF, highly repressed it. Therefore, the AKD9 enzyme was concluded to be a serine protease-type. Furthermore, according to the literature, it was reported that the alkaline protease produced by different species of fungi and bacteria is maximally inhibited by PMSF at 5 mM concentration, indicating that they are serine proteases (Sharma et al., 2019).

### 3.5 *In silico* characterization of AKD9 alkaline serine protease gene

#### 3.5.1 Sequence and phylogenetic analysis of AKD9 gene

**Figure 5:**
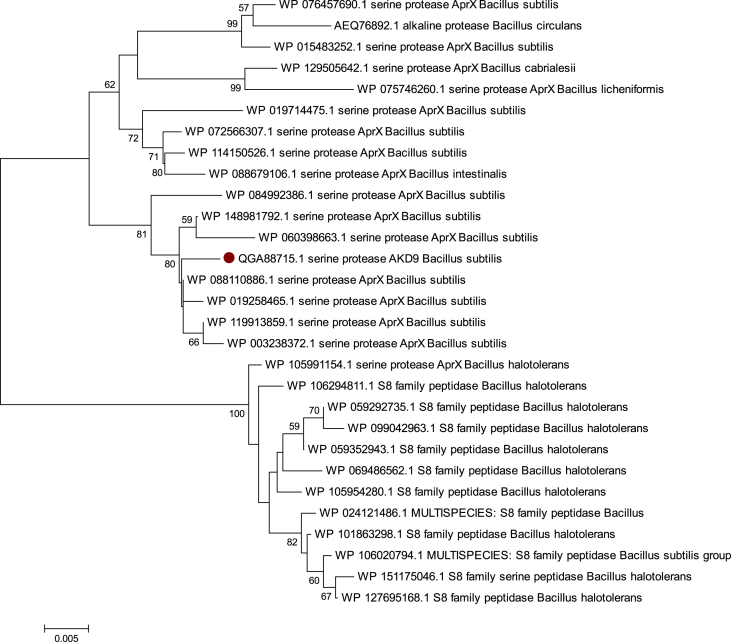
Neighbor-Joining phylogenetic analysis of AKD9 alkaline protease and those of *Bacillus subtilis* group.

*B. subtilis* isolate D9 with high protease productivity was used to amplify the alkaline protease gene AKD9. The deduced amino acid sequence of the alkaline protease gene comprises 442aa, an estimated molecular weight of about 47,789 Da. BLASTp and phylogenetic analysis showed a close relationship between our alkaline protease AKD9 and AprX serine protease of *B. subtilis* isolates (Figure 5).

Together, the high similarity and the close phylogenetic relationship with AprX serine protease of *B. subtilis* isolates confirm that isolate D9 represents isolate of *B. subtilis* and suggest that alkaline protease AKD9 is considered as a serine protease member. Similarly, biochemical analysis revealed a high level of inhibition with serine protease inhibitors confirming that AKD9 is a serine protease.

#### 3.5.2 Functional analysis of AKD9 gene

**Figure 6:**
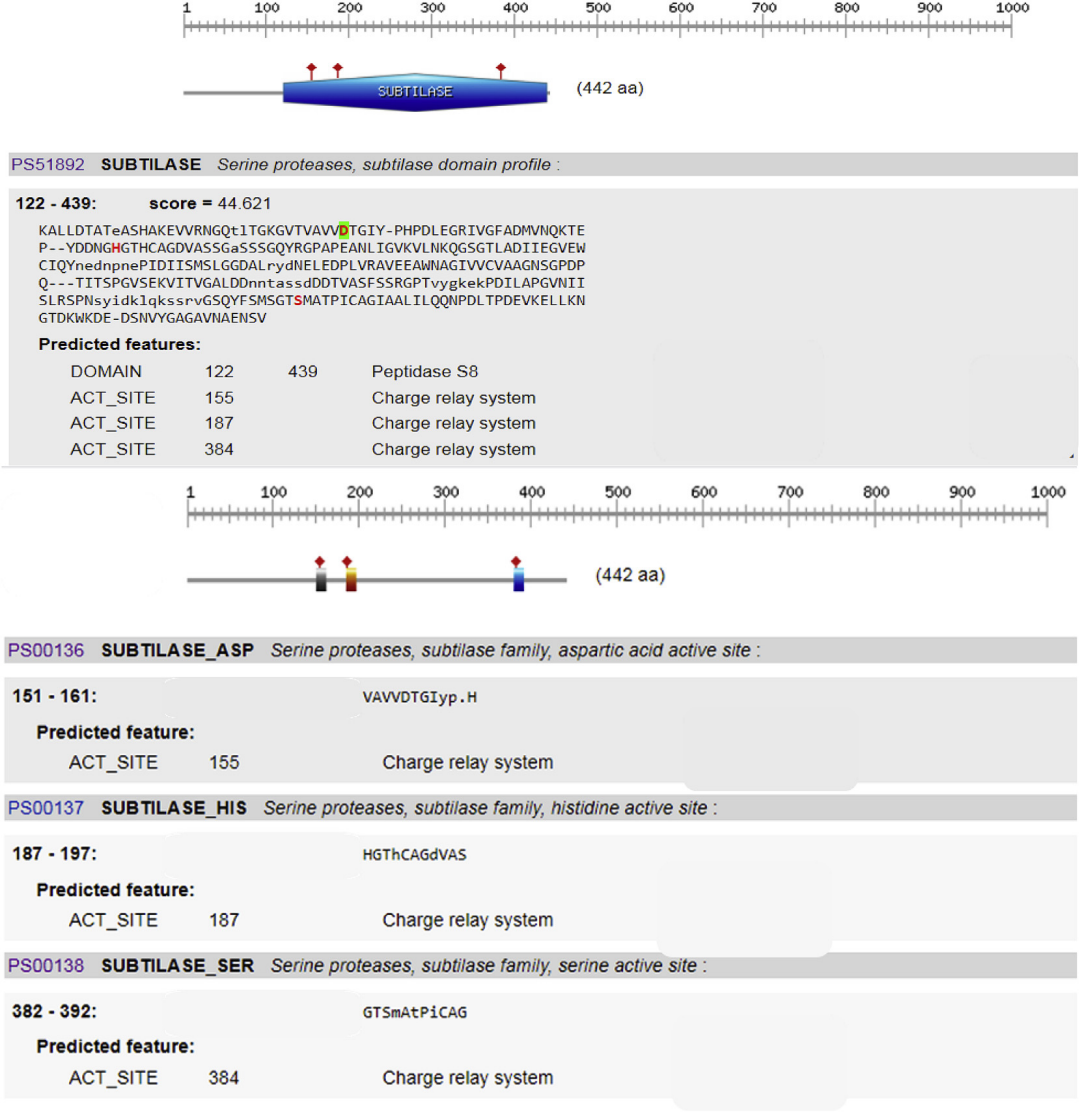
Prediction of the catalytic domain and active sites of serine protease gene AKD9. The catalytic triad subtilase_ASP155, subtilase_HIS187, and subtilase_SER384 were predicted with a score of 44.6.

**Figure 7:**
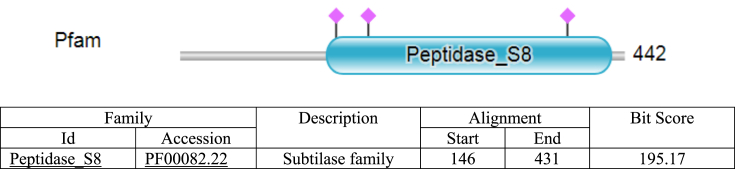
Prediction of AKD9 Peptidase S8 domain subtilase family and the active site using Pfam sequence matches and features compared to Pfam Id Peptidase_S8 PF00082.22. Blue cylinder shape corresponds to peptidase S8 domain subtilase family. Purple diamond shapes correspond to Pfam's predicted active sites D155, H187, and S384.

ScanProsite search tool was used to scan AKD9 serine protease for matches against PROSITE profiles and patterns to identify protein domains, families, and functional sites. Subtilase domain (peptidase S8) was predicted at amino acid position 122–439 of AKD9 (compared to PROSITE entry PS51892), including the catalytic triad subtilase. The catalytic triad subtilase_ASP155, subtilase_HIS187, and subtilase_SER384 were predicted with a score of 44.6 (Figure 6). Similarly, peptidase S8 domain subtilase family and the active site D155, H187 and S384 with score 195.17 were predicted using Pfam sequence matches and features compared to Pfam Id Peptidase_S8 PF00082.22 (Figure 7). It is known that alkaline serine proteases have Aspartate (D) and Histidine (H) residues along with Serine (S) in their active site forming a conserved catalytic triad detected in different microbial serine proteases (Gupta et al., 2002; Raj et al., 2017). Besides, the catalytic triad is considered the main player in the catalytic mechanism in the serine proteases (Gábor et al., 2009).

To confirm the effect of mutations on the predicted functional site, three separate substitutions replaced the catalytic tried as follows: ASP155 was replaced by Serine (S), HIS187 was replaced by Aspartate (D), and SER384 was replaced by Histidine (H). The active site triad substitutions avoided predicting the catalytic tried, thereby confirming the specificity and conservancy of the amino acid of the catalytic triad (supplementary data).

#### 3.5.3 Physicochemical characterization of AKD9 gene

Twelve serine proteases sequences representing different *Bacillus* species were retrieved from NCBI-BLASTp and compared to AKD9 serine protease to better investigate the gene encoding AKD9 protease (Table 2). Different physicochemical characteristics were predicted using Expasy's Protparam server: N amino acids, molecular weight, theoretical *pI*, gravy index, instability index, and aliphatic index, the total number of negatively charged residues of AKD9 alkaline protease (Table 3).

**Table 2:** AKD9-similar serine proteases represent different *Bacillus* species retrieved from BLASTp-NCBI.

No.	Accession number	Species	% identity
1	WP_088110886.1	serine protease AprX [*B. subtilis*]	99.55%
2	WP_088679106.1	serine protease AprX [*B. intestinalis*]	97.29%
3	WP_129505642.1	serine protease AprX [*B. cabrialesii*]	96.61%
4	PWI61024.1	serine protease [*B. subtilis*]	96.15%
5	AEQ76892.1	alkaline protease [*B. circulans*]	95.70%
6	WP_075746260.1	serine protease AprX [*B. licheniformis*]	95.70%
7	WP_087991472.1	serine protease AprX [*B. subtilis*]	94.57%
8	WP_121642617.1	S8 family peptidase [*B.vallismortis*]	93.89%
9	WP_151175046.1	S8 family serine peptidase [*B.halotolerans*]	93.67%
10	WP_029441280.1	S8 family peptidase [*B. mojavensis*]	93.67%
11	WP_024715980.1	S8 family peptidase [*B. tequilensis*]	93.21%
12	WP_106359901.1	S8 family peptidase [*B. atrophaeus*]	89.59%

**Table 3:** Physiochemical properties of AKD9 alkaline serine protease primary structure compared to the similar serine proteases of *Bacillus* species retrieved from GenBank database.

Serine protease		N amino acids	Molecular weight	Asp + Glu/Arg + Lys	Theoretical p*I*	Gravy index	Instability index	Aliphatic index
AKD9 *B. subtilis*		442	47789.68	57/43	5.14	-0.37	30.63	79.84
1	*B. subtilis*	442	47769.69	57/43	5.14	-0.36	30.19	80.07
2	*B. circulans*	442	47857.83	58/43	5.14	-0.37	35.87	79.41
3	*B. subtilis*	442	47875.87	60/43	4.93	-0.34	28.55	81.40
4	*B. licheniformis*	442	47830.79	57/45	5.22	-0.36	32.31	79.64
5	*B. intestinalis*	442	47954.95	59/43	5.05	-0.37	32.31	80.29
6	*B. cabrialesii*	442	47871.75	59/43	5.00	-0.38	33.76	79.64
7	*B. subtilis*	442	47907.83	58/43	5.14	-0.37	33.91	78.53
8	*B.halotolerans*	442	47842.94	58/45	5.28	-0.34	32.89	80.54
9	*B.vallismortis*	442	47847.94	58/45	5.26	-0.34	29.06	81.63
10	*B. tequilensis*	442	47939.14	56/46	5.43	-0.36	29.84	82.96
11	*B. mojavensis*	442	47876.95	58/44	5.16	-0.34	30.51	80.52
12	*B. atrophaeus*	442	48172.20	59/44	5.21	-0.36	28.65	82.08

The molecular weight of the serine proteases ranged from 47.8–48 kDa (47.8 kDa for AKD9), similar to that detected *in vitro* (band below 48 kDa) (Figure 2b). The theoretical isoelectric point (p*I*) is the pH at which a particular molecule or surface carries no net electrical charge; it is useful for understanding the protein charge stability (Gasteiger et al., 2005). The p*I* of the serine proteases ranges from 4.93 to 5.43 (5.14 for AKD9), similar to that detected *in vitro* (p*I* = 5.2) (Figure 4b). The predicted instability index <40 indicates that the protein is stable, while those with values >40 suggest that the protein is unstable (Guruprasad et al., 1990). The instability index value was 28.55–35.87 (30.63 for AKD9), indicating the stability of the serine proteases. The calculated Instability index for APrBL alkaline serine protease of *B. lehensis* was 26.23 (Bhatt and Singh, 2020). A high aliphatic index indicates that the protein is thermostable over a wide temperature range (Ikai, 1980). The high aliphatic index range 78.53–82.96 for the serine proteases and 79.84 for AKD9 indicate its enhanced thermo-stability over a broad range of temperatures. Also, the biochemical analysis indicated that the AKD9 is stable till 65 °C, which can approve the calculated aliphatic index. The number of negatively charged amino acids (Asp + Glu) was higher than the positively charged amino acids (Arg + Lys) for all alkaline serine proteases (Asp + Glu/Arg + Lys was 57/43 forAKD9), indicating alkali-halo stability of the serine proteases. Negative charges are necessary for the tertiary or quaternary structure because they stabilize water and/or ion binding, facilitate protein refolding and prevent aggregation (Takenaka et al., 2011; Bhatt and Singh, 2020). The grand average of hydropathicity (GRAVY) index ranging from -0.38 to -0.34 (−0.37 for AKD9) indicated that AKD9 is a hydrophilic protease and has better interaction with water (Pradeep et al., 2012). As a result, in the industrial sector, protease extraction is easy since it does not bind to the hydrophobic membrane.

#### 3.5.4 Subcellular localization of AKD9 gene

Psortb2 software was used to predict the subcellular localization of the AKD9 serine protease. The protein is extracellular with a Psortb score of 9.6 (Table 4). Some research indicated that *B. subtilis* could produce extracellular protease (Soares et al., 2005; Pant et al., 2015; Blanco et al., 2016).

**Table 4:** Subcellular localization of the AKD9 alkaline serine protease using PSORTb.

Localization	PSORTb score
Cytoplasmic	0.10
Cytoplasmic Membrane	0.14
Cell wall	0.15
Extracellular	9.60
Final Prediction: Extracellular	9.60

#### 3.5.5 Secondary structure

**Figure 8:**
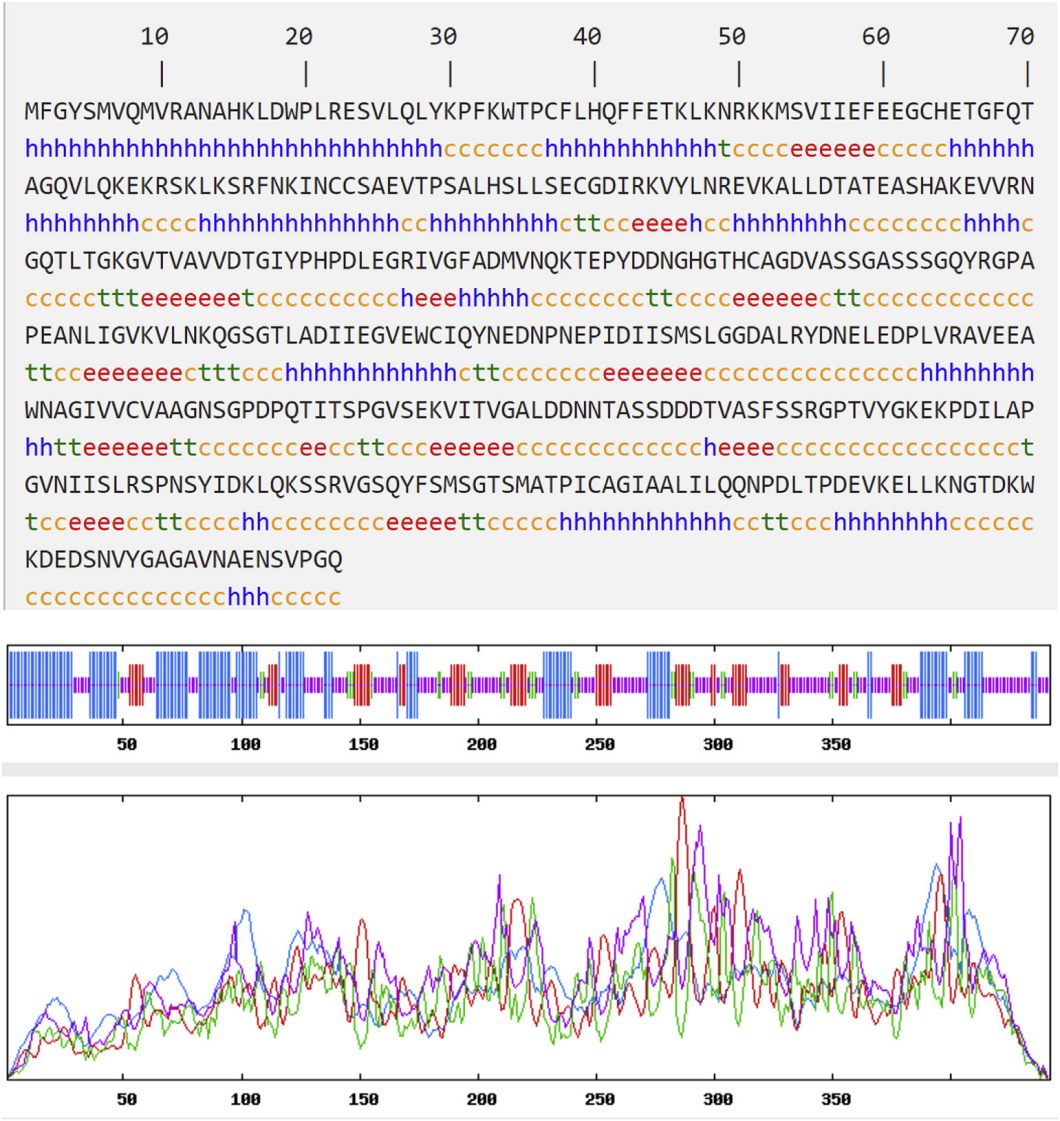
Secondary structure prediction of AKD9 serine protease. Blue h = alphahelix accounted for approximately 32.81%. The red e = beta sheet accounted for approximately 15.16%. The green t = beta turn accounted for approximately 7.24%. The yellow c = random coil accounted for approximately 44.80%.

The secondary structure of AKD9 protease was predicted by applying the SOPMA tool (https://npsa-prabi.ibcp.fr/cgi-bin/npsa_automat.pl?page=/NPSA/npsa_sopma.htmlv). The secondary structure determines whether a given amino acid sequence lies in a helix, strand, or coil (Jyotsna et al., 2010; Ojeiru et al., 2010). The secondary structure of AKD9 serine protease predicted and revealed that AKD9 protease contained 32.81% alpha-helix, 15.16% beta-sheet, 7.24% beta-turn, and 44.80% random coil, indicating the dominance of the coils (Figure 8). The flexible conformation of the coil regions in APrBL alkaline serine protease enhances its activity and stability under the saline and alkaline conditions; structural flexibility in the active site of the enzyme might facilitate efficient substrate binding and catalysis (Bhatt and Singh, 2020). The halophilic and alkaliphilic enzymes need to balance rigidity to prevent the unfolding and flexibility to allow the motions necessary for the catalysis. However, non-halophilic enzymes contain more alpha-helix and beta-sheet forming regions (Zorgani et al., 2014).

#### 3.5.6 Molecular docking

The highest interaction between AKD9 alkaline serine protease and casein recorded a binding score of -71.92 kcal/mol and an RMDS_refine value of 0.70, indicating a very good binding affinity and expected intrinsic activity.

##### 3.5.6.1 Protein-ligand interaction fingerprint (PLIF)

**Figure 9:**
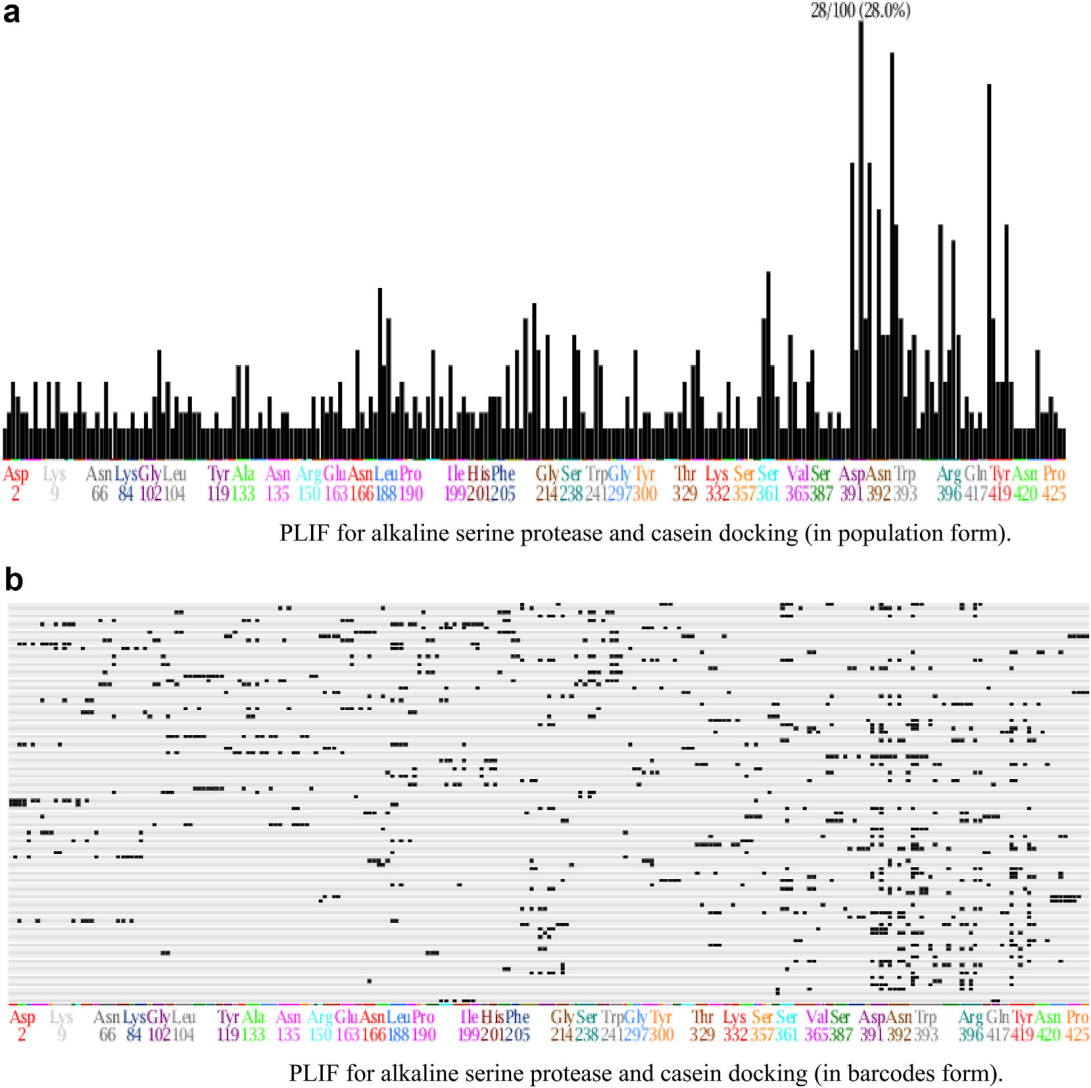
(a) PLIF for alkaline serine protease and casein docking (in population form). (b) PLIF for alkaline serine protease and casein docking (in barcodes form).

The protein-ligand interaction fingerprint (PLIF) was studied for the selected pose from the docking process (pose number 6). It was noted that Asp391 and Asn392 were the most crucial amino acids of the alkaline serine protease (28%) responsible for the binding to casein. Also, Trp393, Thr419, Asn420, Ser361, and Leu188 of the alkaline serine protease formed the most significant portion of interactions (Figure 9a and b).

##### 3.5.6.2 Visualization and description of the selected docking pose

**Figure 10:**
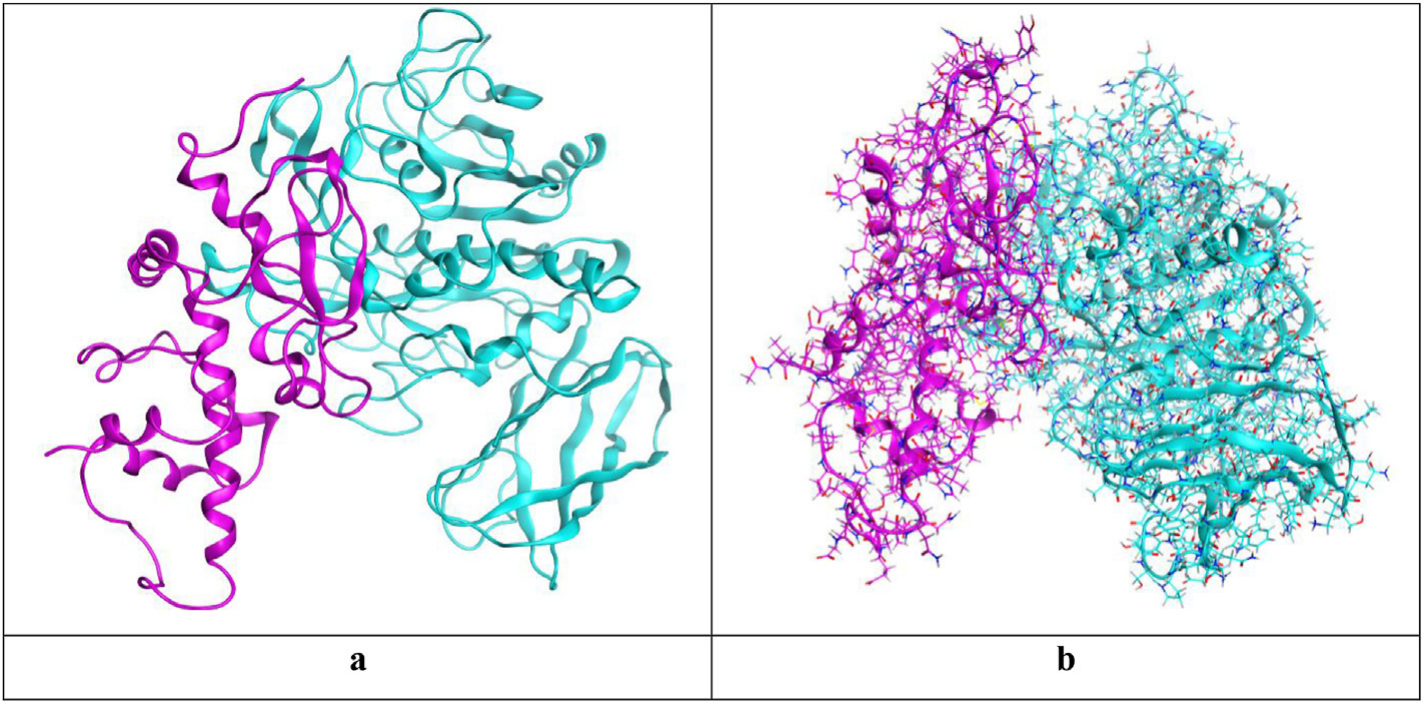
The large surface area of binding interactions between alkaline serine protease and casein (described in turquoise and purple, respectively).

**Figure 11:**
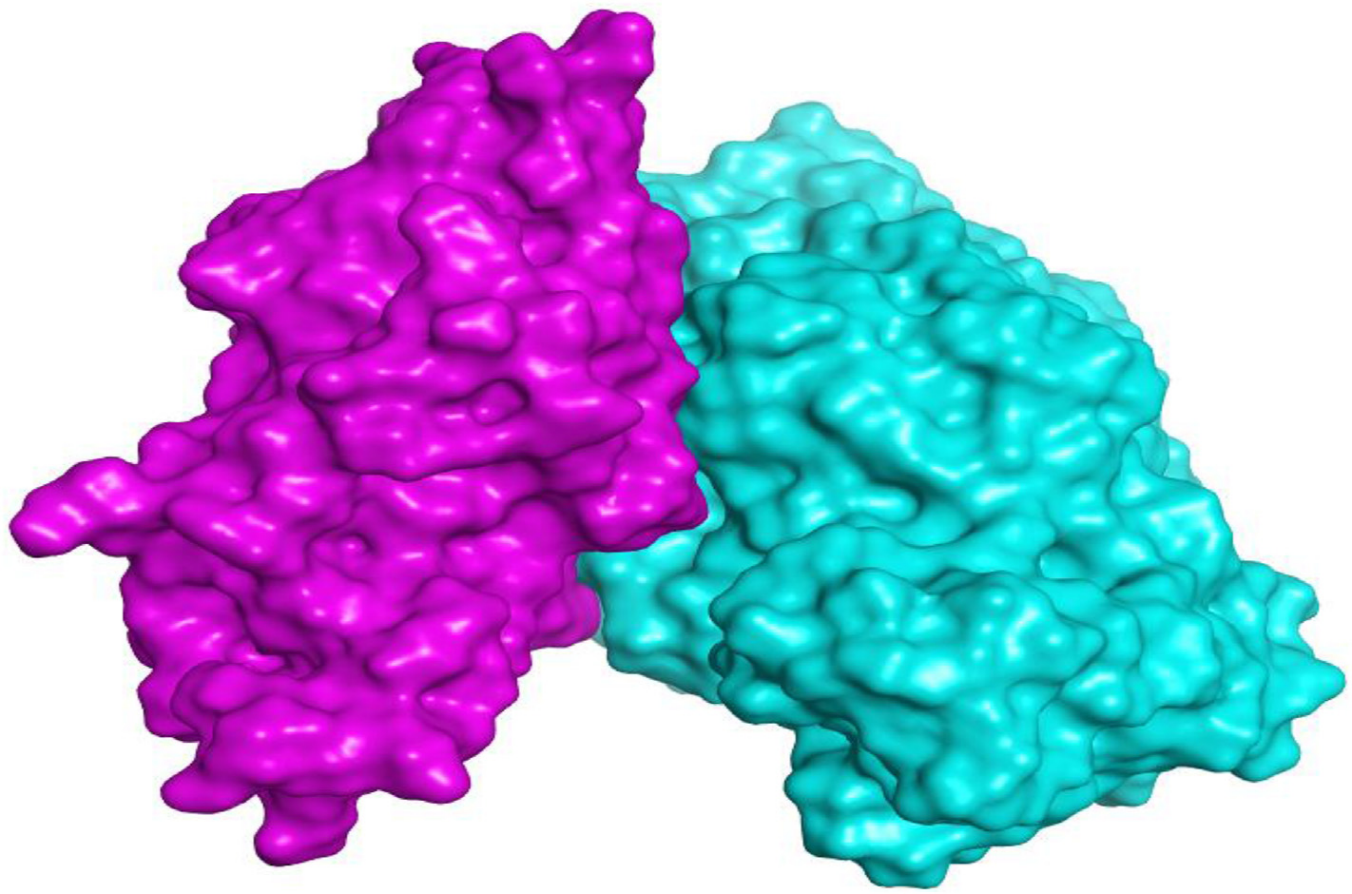
3D binding interactions between alkaline serine protease and casein (described in turquoise and purple, respectively).

The selected docking pose was based on the resulted binding interactions, score, and rmsd_refine value as described earlier. Pose number 6 was selected with a binding score of -64.57 kcal/mol and an RMSD_refine value of 0.66. It is worth mentioning that the interaction between alkaline serine protease and casein occurs through a large surface area greatly recommends the expected intrinsic proteolytic activity as mentioned before (Figures 10a, b and 11).

## 4 Conclusion

The current study focused on the biochemical properties, primary structure, secondary structure, physicochemical properties, protein-protein interactions, and catalytic potential of a *Bacillus subtilis* serine protease. Based on PMSF inhibition of AKD9 enzyme, multiple sequence alignment, phylogenetic and functional prediction, AKD9 protease is classified as alkaline serine proteases and subtilase S8 family. Biochemical analysis showed the stability of AKD9 protease at temperature 65 °C and pH 8–11. In addition, physicochemical properties of the primary structure revealed that AKD9 protease is hydrophilic, thermostable, and alkali-halo stable. Secondary structure analysis confirmed the dominance of the coils enhances AKD9 protease activity and stability under saline and alkaline conditions.

Furthermore, molecular docking of the modeled alkaline serine protease showed very good binding affinities towards the casein substrate. Based on these findings, alkaline serine protease could be a promising candidate for the detergent industry. However, further *in vitro* studies are needed to better understand the effectiveness of AKD9 serine protease as a detergent.

## Declarations

### Author contribution statement

Amal Mahmoud, Essam Kotb: Conceived and designed the experiments; Analyzed and interpreted the data; Contributed reagents, materials, analysis tools or data; Wrote the paper.

Amany I. Alqosaibi, Ibtesam S. Al-Dhuayan, Hameedah Alabkari: Performed the experiments; Contributed reagents, materials, analysis tools or data; Wrote the paper.

Ahmed A. Al-Karmalawy: Analyzed and interpreted the data; Contributed reagents, materials, analysis tools or data; Wrote the paper.

### Funding statement

This work was supported by the Deanship of Scientific Research, 10.13039/501100015089Imam Abdulrahman Bin Faisal University, Kingdom of Saudi Arabia for funding (project number Sci-113-2017).

### Data availability statement

Data associated with this study has been deposited at GenBank under the accession numbers MK819971–MK819982.

### Competing interest statement

The authors declare no conflict of interest.

### Additional information

No additional information is available for this paper.

## References

[bib1] Abo Elmaaty A., Darwish K.M., Khattab M., Elhady S.S., Salah M., Hamed M.I.A., Al-Karmalawy A.A., Saleh M.M. (2021). In a search for potential drug candidates for combating COVID-19: computational study revealed salvianolic acid B as a potential therapeutic targeting 3CLpro and spike proteins. J. Biomol. Struct. Dyn..

[bib2] Al-Dhuayan I., Kotb E., Alqosaibi A., Mahmoud A. (2021). Histological studies on a newly isolated Bacillus subtilis D10 protease in the debridement of burn wound eschars using mouse model. Pharmaceutics.

[bib3] Andrews A.T. (1986).

[bib4] Baweja M., Tiwari R., Singh P.K., Nain L., Shukla P. (2016). An alkaline protease from Bacillus pumilus MP 27: functional analysis of its binding model toward its applications as detergent additive. Front. Microbiol..

[bib5] Bhatt H.B., Singh S.P. (2020). Cloning, expression, and structural elucidation of a biotechnologically potential alkaline serine protease from a newly isolated haloalkaliphilic Bacillus lehensis JO-26. Front. Microbiol..

[bib6] Blanco A.S., Durive O.P., Pérez S.B., Montes Z.D., Guerra N.P. (2016). Simultaneous production of amylases and proteases by Bacillus subtilis in brewery wastes. Braz. J. Microbiol..

[bib7] Chanalia P., Gandhi D., Jodha D., Singh J. (2011). Applications of microbial proteases in pharmaceutical industry: an overview. Rev. Med. Microbiol..

[bib8] Chantalat L., Leroy D., Filhol O., Nueda A., Benitez M.J., Chambaz E.M., Cochet C., Dideberg O. (1999). Crystal structure of the human protein kinase CK2 regulatory subunit reveals its zinc finger-mediated dimerization. EMBO J..

[bib9] Clarridge J.E. (2004). Impact of 16S rRNA gene sequence analysis for identification of bacteria on clinical microbiology and infectious diseases. Clin. Microbiol. Rev..

[bib12] Dash S.G., Maharana J.K., Patel A.K. (2017). Microbial characterization and optimization of protease production by Bacillus S.P. Isolated from soil. Int. J. Inf. Educ. Technol..

[bib13] El shal M.F., Eid N.M., El-Sayed I., El-Sayed W., Al-Karmalawy A.A. (2021).

[bib14] Elhefnawi M.M., Hasan M.E., Mahmoud A., Khidr Y.A., El-Absawy E.A., Hemeida A.A. (2019). Prediction and analysis of three dimensional structure of the p7-transactivated protein1 of hepatitis C virus. Infect. Disord. - Drug Targets.

[bib15] Ellaiah P., Srinivasulu B., Adinarayana K. (2002). A review on microbial alkaline proteases. J. Sci. Ind. Res..

[bib16] Fath M., Fazaelipoor M.H. (2015). Production of proteases in a novel trickling tray bioreactor. Waste Bioma. Valoriz.

[bib17] Gábor I., Zoltán S., Rafael O., Vince G., Gábor N.-S. (2009). Four spatial points that define enzyme families. Biochem. Biophys. Res. Commun..

[bib18] Gasteiger E., Hoogland C., Gattiker A., Walker J.M. (2005). The Proteomics Protocols Handbook.

[bib19] Geourjon C., Deleage G. (1995). SOPMA: significant improvements in protein secondary structure prediction by consensus prediction from multiple alignments. Bioinformatics.

[bib20] Gupta R., Beg Q.K., Lorenz P. (2002). Bacterial alkaline proteases: molecular approaches and industrial applications. Appl. Microbiol. Biotechnol..

[bib21] Guruprasad K., Reddy B., Pandit M.W. (1990). Correlation between stability of a protein and its dipeptide composition: a novel approach for predicting in vivo stability of a protein from its primary sequence. Protein Eng..

[bib22] Ikai A. (1980). Thermostability and aliphatic index of globular proteins. J. Biochem..

[bib23] Inc (2016).

[bib24] Jyotsna C., Ashish P., Shailendra G., Verma M.K. (2010). Homology modeling and binding site identification of 1 deoxy d- xylulose 5 phosphate reductoisomerase of plasmodium falciparum, new drug target for plasmodium falciparum. Int. J. Eng. Sci. Technol..

[bib25] Kandasamy S., Senbagam D., Chinnappan S., Senthilkumar B., Selvankumar T., Govarthanan M., Arumugam S., Palanisamy S. (2017). Molecular modeling and docking of protease from Bacillus sp. for the keratin degradation. Biocatalys. Agricult. Biotechn..

[bib26] Karray A., Alonazi M., Horchani, Ben H., Bacha A. (2021). A novel thermostable and alkaline protease produced from Bacillus stearothermophilus isolated from olive oil mill sols suitable to industrial Biotechnology. Molecules.

[bib27] Kotb E. (2015). The biotechnological potential of subtilisin-like fibrinolytic enzyme from a newly isolated *Lactobacillus Plantarum* KSK-II in blood destaining and antimicrobials. Biotechnol. Prog..

[bib28] Li J., Chi Z., Wang X., Peng Y., Chi Z. (2009). The selection of alkaline protease-producing yeasts from marine environments and evaluation of their bioactive peptideproduction. Chin. J. Oceanol. Limnol..

[bib29] Madala P.K., Tyndall J.D., Nall T., Fairlie D.P. (2010). Update 1 of: proteases universally recognize beta strands in their active sites. Chem. Rev..

[bib30] Marks D.S., Hopf T.A., Sander C. (2012). Protein structure prediction from sequence variation. Nat. Biotechnol..

[bib31] Maughan H., Van der Auwera G. (2011). Bacillus taxonomy in the genomic era finds phenotypes to be essential though often misleading. Infect. Genet. Evol..

[bib32] Mohkam M., Nezafat N., Berenjian A. (2016). Identification of Bacillus probiotics isolated from soil rhizosphere using 16S rRNA, recA, rpoB gene sequencing and RAPD-PCR. Probiotics Antimicrob Proteins.

[bib33] Morya V.K., Yadav S., Kim E.-K., Yadav D. (2012). In *silico* characterization of alkaline proteases from different species of Aspergillus. Appl. Biochem. Biotechnol..

[bib34] Nonaka T., Fujihashi M., Kita A., Saeki K., Ito S., Horikoshi K., Miki K. (2004). The crystal structure of an oxidatively stable subtilisin-like alkaline serine protease, KP-43, with a C-terminal beta-barrel domain. J. Biol. Chem..

[bib35] Ojeiru F.E., Kazuya T., Yuki H., Mohammed S.M., Shunsuke M. (2010). Circular dichroism studies on C-terminal zinc finger domain of transcription factor GATA-2. Yonago Acta Med..

[bib36] Page M.J., Di Cera E. (2008). Serine peptidases: classification, structure and function. Cell. Mol. Life Sci..

[bib37] Pant G. (2015). Production, optimization and partial purification of protease from *Bacillus subtilis*. J Taibah Univ Sci.

[bib38] Paul L.P., Thangamani R., Paramasamy (2011). Identification and characterization of alkaline serine protease from goat skin surface metagenome. Amb. Express.

[bib39] Pradeep N.V., Anupama1 Vidyashree K.G., Lakshmi P. (2012). In silico characterization of industrial important cellulases using computational tools. Adv. Life Sci. Technol..

[bib40] Pushpam P.L., Rajesh T., Gunasekaran P. (2011). Identification and characterization of alkaline serine protease from goat skin surface metagenome. Amb. Express.

[bib41] Raj T., Sharma N., Savitri (2017). Bacterial serine proteases: computational and statistical approach to understand temperature adaptability proteomics. Bioinformation.

[bib42] Raveendran S., Parameswaran B., Beevi U.S. (2018). Applications of microbial enzymes in food industry. Food Technol. Biotechnol..

[bib43] Rawlings N.D., Morton F.R., Barrett A.J. (2006). MEROPS: the peptidase database. Nucleic Acids Res..

[bib44] Rawlings N.D., Barrett A.J., Bateman A. (2010). MEROPS: the peptidase database. Nucleic Acids Res..

[bib45] Razzaq A., Shamsi S., Ali A., Ali Q., Sajjad M. (2019). Microbial proteases applications. Front. Bioeng. Biotechnol.

[bib46] Sadeghi M.M., Rabbani M., Naghitorabi M. (2009). Cloning of alkaline protease gene from *Bacillus subtilis* 168 H. Res. Pharama. Sci..

[bib47] Saggu S.K., Mishra P.C. (2017). Characterization of thermostable alkaline proteases from Bacillus infantis SKS1 isolated from garden soil. PLoS One.

[bib48] Samra R.M., Soliman A.F., Zaki A.A., Ashour A., Al-Karmalawy A.A., Hassan M.A., Zaghloul A.M. (2021). Bioassay-guided isolation of a new cytotoxic ceramide from Cyperus rotundus L. South Afr. J. Bot..

[bib49] Sharma M., Gat Y., Arya S., Kumar V., Panghal A., Kumar A. (2019). A review on microbial alkaline protease: an essential tool for various industrial approaches. Ind. Biotechnol..

[bib50] Sigrist C.J.A., de Castro E., Cerutti L. (2013). New and continuing developments at PROSITE. Nucleic Acids Res..

[bib51] Soares V.F., Castilho L.R., Bon E.P. (2005). Twenty-Sixth Symposium on Biotechnology for Fuels and Chemicals.

[bib52] Srinivasan R., Karaoz U., Volegova M. (2015). Use of 16S rRNA gene for identification of a broad range of clinically relevant bacterial pathogens. PLoS One.

[bib53] Takenaka S., Yoshida N., Yoshida K.I., Murakami S., Aoki K. (2011). Molecular cloning and sequence analysis of two distinct halotolerant extracellular proteases from Bacillus subtilis FP-133. Biosci. Biotechnol. Biochem..

[bib54] Tamura K., Stecher G., Peterson D. (2013). MEGA6: molecular evolutionary genetics analysis version 6.0. Mol. Biol. Evol..

[bib60] Tarhriz V., Hamidi A., Rahimi E. (2014). Isolation and characterization of naphthalene degradation bacteria from Qurugol lake located at Azerbaijan. Biosci. Biotechnol. Res. Asia..

[bib56] Yu N.Y., Wagner J.R., Laird M.R. (2010). PSORTb 3.0: improved protein subcellular localization prediction with refined localization subcategories and predictive capabilities for all prokaryotes. Bioinformatics.

[bib57] Zhang Z., Hao H., Tang Z. (2015). Identification and characterization of a new alkaline thermolysin-like protease, BtsTLP1, from Bacillus thuringiensis serovar sichuansis strain MC28. J. Microbiol. Biotechnol..

[bib58] Zhou C., Qin H., Chen X. (2018). A novel alkaline protease from alkaliphilic Idiomarina sp. C9-1 with potential application for eco-friendly enzymatic dehairing in the leather industry. Sci. Rep..

[bib59] Zorgani M.A., Patron K., Desvaux Ml (2014). New insight in the structural features of haloadaptation in a-amylases from halophilic Archaea following homology modeling strategy: folded and stable conformation maintained through low hydrophobicity and highly negative charged surface. J. Comput. Aided Mol. Des..

