# Supplementary material for: In vitro and in silico characterization of alkaline serine protease from Bacillus subtilis D9 recovered from Saudi Arabia
Source: Heliyon. 2021 Oct 8;7(10):e08148. doi: 10.1016/j.heliyon.2021.e08148 (PMC8524146; doi:10.1016/j.heliyon.2021.e08148)
Supplement: Supplement _Figure 1 [file mmc1.docx]

**Supplementary data**


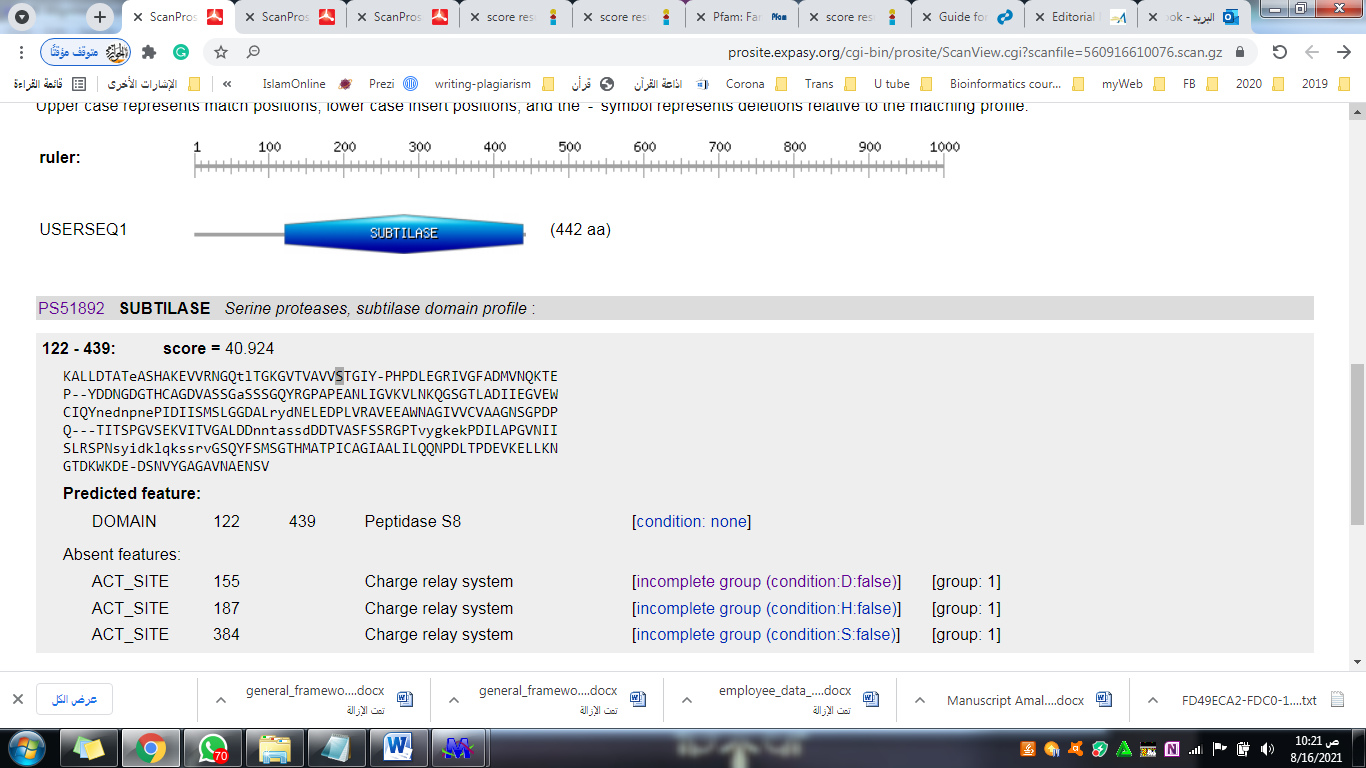


**Figure S1.** The effect of mutations on the predicted functional site, 3 separate substitutions replaced the catalytic tried as follow: ASP155 was replaced by Serine (S), HIS187 was replaced by Aspartate (D) and SER384 was replaced by Histidine (H). The active site triad substitutions avoided predicting the catalytic tried, thereby confirming the specificity and conservancy of the amino acid of the catalytic triad.
